# Nicotinic Acid as a Phosphate-lowering Agent in Patients with End-stage Renal Disease on Maintenance Hemodialysis: A Single-center Prospective Study

**DOI:** 10.7759/cureus.4566

**Published:** 2019-04-30

**Authors:** Syed A Khalid, Faisal Inayat, Muhammad K Tahir, Aamna Younus, Hafiz Ijaz Ahmad, Syed Rizwan A Bokhari, Usman Yaqoob

**Affiliations:** 1 Nephrology, Allama Iqbal Medical College, Lahore, PAK; 2 Internal Medicine, Allama Iqbal Medical College, Lahore, PAK; 3 Internal Medicine, Shifa College of Medicine, Islamabad, PAK; 4 Nephrology, Tulane University School of Medicine, New Orleans, USA; 5 Gastroenterology, Mayo Clinic, Rochester, USA

**Keywords:** nicotinic acid, end-stage renal disease, hyperphosphatemia, serum phosphorus, hemodialysis

## Abstract

Background

Hyperphosphatemia increases the risk of mortality and morbidity in patients with end-stage renal disease (ESRD). In addition to dietary restriction and renal replacement therapy, phosphorus-binding agents are the mainstay of treatment. While the use of calcium-containing binders has certain limitations, non-calcium-based binders are expensive and not readily available in developing countries. Previous studies on nicotinic acid as a phosphorus-lowering agent have limited data. In this study, we evaluated the efficacy of nicotinic acid in patients with ESRD on hemodialysis (HD) in Pakistan.

Methods

Forty-five patients with ESRD on maintenance HD having serum phosphorus levels >5.5 mg/dL were recruited. Nicotinic acid 250 mg was administered with food for four weeks. All patients with serum phosphorus levels <8 mg/dL were placed on a twice-daily regimen while the rest received it three times a day with meals. Patients were assessed at the beginning and end of the study with serum phosphorus levels.

Results

Mean age of the sample population was 44.6 ± 13.9 years and 57.8% of participants were male. Serum phosphorus level before treatment ranged from 5.6 to 10.8 mg/dL (mean, 6.91 ± 1.33). After nicotinic acid therapy, it ranged from 2.60 to 8.70 mg/dL (mean, 5.82 ± 1.40). Mean decrease in serum phosphorus levels with nicotinic acid after one month of treatment was 1.08 ± 1.16 mg/dL (p-value <0.001).

Conclusion

Nicotinic acid is effective in lowering serum phosphorus levels in patients with ESRD who are under renal replacement therapy with* *maintenance HD.

## Introduction

The global incidence of end-stage renal disease (ESRD) is increasing exponentially. By 2030, the number of individuals on dialysis is projected to reach 5.4 million, with the most growth expected in Asia [1]. Hyperphosphatemia is a potential cause of adverse clinical outcomes in ESRD. In patients on maintenance hemodialysis (HD), the mortality risk increases up to 27% with serum phosphorus levels >6.5 mg/dL compared to serum phosphorus levels ranging from 2.4 to 6.5 mg/dL [2]. Similarly, dismal results have been observed for calcium-phosphorus (Ca-P) product of >45.9 mg2/dL2. Chronically elevated calcium has a predilection for precipitation in myocardium, blood vessels, and heart valves [3]. Therefore, appropriate treatment of hyperphosphatemia in patients with ESRD is imperative to decrease the risk of cardiovascular morbidity as well as renal complications, such as renal failure and renal osteodystrophy [4].

In prior research, nicotinic acid has been demonstrated to be safe, cheap, and effective in normalizing serum phosphorus and Ca-P product in patients with ESRD [5]. Flushing, a common side effect of nicotinic acid, can be overcome by premedication with aspirin [6]. Although most side effects are reversible, further studies are warranted to investigate the safety profile of this potential therapeutic option [7]. We conducted this prospective study to evaluate the relative efficacy of nicotinic acid as a cost-effective, non-calcium-containing phosphate binder at a relatively low dose in patients with ESRD on maintenance HD in Pakistan. The preliminary form of the data was presented as an abstract (Abstract: Bokhari SRA, Tahir MK, Ahmad HI, Asif A. Role of nicotinic acid as a phosphate-lowering agent in end-stage renal disease on maintenance hemodialysis, American Society of Nephrology Kidney Week; November 03-08, 2015, San Diego, California).

## Materials and methods

Patients

This prospective study was conducted at the Hemodialysis Center, Department of Nephrology, Jinnah Hospital, Allama Iqbal Medical College, Lahore. The project was completed in a period of six months from September 03, 2014 until March 02, 2015. Non-probability consecutive sampling was followed for the recruitment of the participants. A total of 56 patients admitted in our department were screened for this study. The patients with age ranging from 20 to 65 years who were on maintenance HD for more than three months, receiving a stable dose of phosphate binders (calcium carbonate/calcium gluconate) during the last two weeks, and had serum phosphorus levels >5.5 mg/dL in the last one month, were included in the study. The patients undergoing any chemotherapy and those with a history of autoimmune disorder, peptic ulcer disease, liver disease as well as pregnant women were excluded.

Study design

Forty-five patients with ESRD fulfilled the recruitment criteria. The participants underwent thrice weekly HD, four hours for each session on Toray TQS 88 machines with Fresenius Fx8 dialyzers (Fresenius Medical Care, Germany); blood flow ranging from 300-350 mL/minute with the dialysate flow rate of 500 mL/minute. With regard to the data collection, specially designed and authenticated proformas were used. The data collection sheet included variables such as the duration on HD, the frequency of HD per week, and measurements of serum phosphorus levels, and Ca-P product at baseline and end of the study. Nicotinic acid at a dose of 250 mg was administered with meals for four weeks. The patients with serum phosphorus levels of 5.1-7.9 mg/dL received twice a day, whereas those with serum phosphorus levels of >7.9 mg/dL were placed on a thrice-a-day dose. A 5-mL pre-dialysis blood sample was drawn from each patient on the first HD of the week in the beginning and end of the study duration. Serum phosphorus levels were measured at the baseline and compared after four weeks of nicotinic acid treatment to assess the mean decrease in serum phosphorus.

Ethics and consent

The research protocol was approved by the Institutional Review Board of Allama Iqbal Medical College, Jinnah Hospital, Lahore. All patients granted written informed consent before participating in the study, which was conducted according to the principles of the Declaration of Helsinki as revised in Seoul in 2008.

Statistical analysis

The data analysis was performed using SPSS 21 statistical package (IBM SPSS, Inc., Chicago, Illinois). Qualitative variables such as age etc. were presented as frequency and percentage while quantitative variable, including serum phosphorus levels, were described as mean ± standard deviation (SD). Data was stratified for age and gender. Mean decrease in serum phosphorus levels before and after treatment was compared using paired sample t-test. Independent sample t-test was used to compare the mean difference in serum phosphorus level before and after treatment in male and female participants, respectively. A p-value of <0.05 was considered significant.

## Results

A total of 45 patients were included in the final analysis. The mean age of patients was 44.60 ± 13.975 years (range: 23 to 76 years). In our study sample, (n = 45), 26 patients (57.8%) were male while remaining 19 patients (42.2%) were female. Sampled population (n = 45) was categorized into two groups according to the age. Nineteen patients (42.2%) were either 40 years or below, whereas the remaining 26 patients (57.8%) were above 40 years of age (Table 1).

**Table 1:** Gender and age distribution of the sample patient population

Patient characteristics	Number (%)
Male	26 (57.8%)
Female	19 (42.2%)
40 years and below	19 (42.2%)
Above 40 years	26 (57.8%)
Mean age	44.6 ± 13.97 years

Serum phosphorus levels before treatment ranged from 5.6 to 10.8 mg/dL with mean of 6.911 ± 1.3328 mg/dL. After treatment, it ranged from 2.60 to 8.70 mg/dL with a mean value of 5.8267 ± 1.408 mg/dL. Mean difference was 1.08 ± 1.166 mg/dL. The difference between serum phosphorus levels, before treatment and after treatment, showed significant results (p = 0.000) (Table 2).

**Table 2:** Analysis of the mean phosphorus levels before and after therapy, using paired sample t-test (p-value <0.001)

Serum phosphorus levels	Mean and standard deviation
At the initiation of therapy	6.911 ± 1.3328
After 4 weeks of therapy	5.8267 ± 1.40816
The difference after 4 weeks	1.08 ± 1.166

We cross-tabulated the gender with the difference in the serum phosphorus levels after treatment to determine the distribution difference in serum phosphorus levels after treatment among male and female participants, which showed non-significant results on independent samples test (p = 0.959) (Table 3).

**Table 3:** Analysis of gender distribution of the difference in phosphorus levels after therapy using independent samples t-test and assuming equal variances (p-value = 0.959)

Serum phosphorus levels	Gender	Frequency	Mean and standard deviation
The difference after 4 weeks	Male	26	1.09 ± 1.962
	Female	19	1.07 ± 1.203

When we cross-tabulated age groups with a difference in serum phosphorus levels after treatment, the difference was not significant (p = 0.460). Nineteen patients with a mean of 1.24 ± 1.152 mg/dL were either 40 years or below age; however, rest of 26 with the mean of 0.97 ± 1.185 mg/dL were above 40-year-old (Table 4).

**Table 4:** Analysis of age distribution of the difference in phosphorus levels after therapy using independent samples t-test and assuming equal variances (p-value = 0.46)

Serum phosphorus levels	Age group (years)	Frequency	Mean and standard deviation
The difference after 4 weeks	40 and below	19	1.24 ± 1.152
	Above 40	20	0.97 ± 1.185

## Discussion

The use of phosphate-lowering agents is indicated to optimize serum phosphorus levels in patients with ESRD as dietary phosphorus restriction and dialysis are rarely sufficient [8]. These agents are classified into calcium-based and non-calcium-based phosphate binders. In patients with hyperphosphatemia, the use of calcium-based agents has demonstrated significant improvement in serum phosphorus levels [9]. However, randomized controlled trials on calcium acetate and calcium carbonate highlighted an impending risk of hypercalcemia-related vascular calcification and coronary atherosclerosis [10]. Additionally, calcium-based binders are avoided in patients with combined hyperphosphatemia and hypercalcemia. Sevelamer is a non-calcium-based binder with similar effects on hyperphosphatemia and lower risk of hypercalcemia-associated morbidity, even in patients who were previously treated with calcium-based binders [11]. Lanthanum is also an effective non-calcium-based binder that increases compliance by reducing the total pill burden. However, non-calcium-based binders are expensive and relatively high treatment cost constitutes a major limiting factor in their use, especially in developing countries.

Nicotinic acid, a non-calcium-based agent, has recently been studied as a new and economical therapeutic option for hyperphosphatemia in patients with ESRD [12]. It inhibits the sodium-phosphorus (Na-Pi) cotransport in kidneys and intestine. It works mainly by limiting gastrointestinal phosphate absorption. Several studies highlighted the role of nicotinic acid in controlling serum phosphorus levels but were limited by small sample size, uncontrolled design, or short duration of treatment. A recent randomized controlled trial using low-dose nicotinic acid on 70 patients with ESRD demonstrated promising results with a mean decrease in phosphorus levels from 6.7 mg/dL (after four weeks of therapy) to 4.4 mg/dL (after 12 weeks of therapy) (p = 0.004) [13]. Furthermore, a significant decrease in phosphorus levels in patients on HD has also been reported [14-15]. Nicotinic acid is similar in efficacy to lanthanum in lowering phosphate levels; however, an optimal dose remains to be determined [16].

In this study, we employed nicotinic acid at a dose of 250 mg, which is relatively lower in comparison to most studies published in the medical literature. The decrease in serum phosphorus levels (1.08 ± 1.16) after four weeks of nicotinic acid therapy was significant when we applied paired sample t-test (p-value <0.001). The difference reported in our study was lower than that reported by Vasantha et al. in 30 dialysis patients receiving nicotinamide. They found a mean decrease of 2.3 mg/dL in serum phosphorus levels after eight weeks of treatment [17]. Shahbazian et al. conducted a study in which 48 dialysis patients received nicotinamide and found a decrease in serum phosphate levels from 5.9 to 4.77 mg/dL after four weeks of treatment [18]. Therefore, our findings add clinical evidence to the existing literature that nicotinic acid can be effectively used as a phosphate-lowering agent in patients with ESRD on HD. Of note, the mean age of participants in our study was approximately 45 years. This relatively younger population emphasizes the need for an extensive ESRD prevention program in this region.

Results from larger and more significant trials emphasize that the safety profile of nicotinic acid is controversial [19]. A sub-group analysis on a randomized controlled Atherothrombosis Intervention in Metabolic Syndrome with low HDL/High Triglycerides: Impact on Global Health Outcomes (AIM-HIGH) trial suggested more side effects (flushing) and fewer benefits resulting from nicotinic acid therapy [20]. The study enrolled 3414 cases and used extended-release niacin. A study comparing sevelamer and oral nicotinamide in 100 patients on HD suggested more side effects (major risk of thrombocytopenia) and less clinical effectiveness of nicotinamide [21]. Concrete evidence for regular use of niacin in HD as a stand-alone therapy still needs extensive studies. The current study is limited by purposive sampling, uncontrolled design, lack of blinding and small sample size; however, it relays useful information on ESRD management pertinent to patient populations in the developing countries.

## Conclusions

This study implicates nicotinic acid as effective in reducing serum phosphorus levels in patients with ESRD on maintenance HD. Addition of low-cost niacin to the treatment regimens of advanced ESRD in developing countries will be of significant benefit in terms of hyperphosphatemia-related morbidity and mortality. In the future, larger, more controlled, prospective, and longitudinal trials are warranted to validate these findings and investigate the adverse events associated with nicotinic acid therapy.
